# Minimizing incisional hernia: intracorporeal anastomosis makes the difference after laparoscopic right colectomy

**DOI:** 10.1007/s00384-025-04903-z

**Published:** 2025-05-08

**Authors:** Ernesto De Giulio, Giulia Turri, Ruben Sciortino, Matteo Rivelli, Gabriele Gecchele, Alessandro Valdegamberi, Tommaso Campagnaro, Andrea Ruzzenente, Corrado Pedrazzani

**Affiliations:** 1 Division of General and Hepatobiliary Surgery, Department of Surgical Sciences, Dentistry, Gynecology and Pediatrics, University of Verona Hospital Trust, University of Verona, Verona, Italy; 2 Division of General and Hepatobiliary Surgery, Department of Engineering for Innovation Medicine, University of Verona, Verona, Italy

**Keywords:** Colon cancer, Laparoscopic right colectomy, Intracorporeal anastomosis, Incisional hernia

## Abstract

**Purpose:**

The anastomosis technique following laparoscopic right colectomy remains a subject of ongoing debate. One of the potential advantages of intracorporeal anastomosis is the flexibility it offers in selecting the location of the minilaparotomy. This study aimed to evaluate the differences in the rate of incisional hernia between intracorporeal and extracorporeal anastomosis after laparoscopic right colectomy.

**Methods:**

We retrospectively analysed patients undergoing laparoscopic right colectomy for colon neoplasia between April 2013 and January 2024, retrieved from a prospectively maintained database. The occurrence of incisional hernia was assessed according to the anastomosis technique. Univariate and multivariate analyses were performed to investigate the relationship between incisional hernia and anastomosis technique, while controlling for other risk factors.

**Results:**

Among 192 patients, 94 underwent intracorporeal anastomosis and 98 underwent extracorporeal anastomosis. The groups were comparable in terms of clinical, pathological, and surgical data. The intracorporeal group showed a lower incidence, although not statistically significant, of postoperative ileus (*p* = 0.052), and a shorter hospital stay (*p* = 0.003). No incisional hernias were observed at the minilaparotomy site in the intracorporeal anastomosis group, while 13.3% of patients in the extracorporeal anastomosis group developed an incisional hernia (*p* < 0.001). One incisional hernia at the umbilical trocar site occurred after intracorporeal anastomosis. Multivariate analysis identified postoperative general complications (OR [95% CI]: 4.1 [1.0–16.5], *p* = 0.049) and extracorporeal anastomosis (OR [95% CI]: 15.4 [1.0–126.9], *p* = 0.011) as independent risk factors for incisional hernia.

**Conclusions:**

Intracorporeal anastomosis significantly reduces the incidence of incisional hernia at the minilaparotomy site. This finding is further supported by logistic regression analysis, which identified intracorporeal anastomosis as a significant and independent protective factor against incisional hernia.

## Introduction

Minimally invasive surgery (MIS) has become the standard for colorectal cancer treatment [1]. Compared to conventional open surgery, MIS offers significant advantages in postoperative recovery while maintaining equivalent oncological outcomes [2, 3]. Patients benefit from smaller incisions, reduced hospital stay, and a lower incidence of wound-related complications, such as surgical site infection (SSI) and incisional hernia (IH) [4, 5]. Laparoscopic surgery has markedly decreased the risk of IH, a severe long-term complication that significantly impairs patients’ quality of life and physical function while increasing healthcare costs [6, 7]. Nevertheless, the risk of extraction-site IH remains not negligible, with reported incidence ranging from 3.2% to 13% following laparoscopic colorectal surgery [8, 9].

Despite its widespread adoption, laparoscopic technique has not been fully standardized, particularly concerning the method of anastomosis following laparoscopic right colectomy (LRC), which, in case of extracorporeal anastomosis (ECA), dictates the site of specimen extraction [10–12]. Intracorporeal anastomosis (ICA) is technically more challenging and requires advanced suturing skills. Furthermore, it may be associated with an increased risk of infection due to faecal spillage, potentially leading to both superficial and deep SSIs [13, 14]. However, ICA provides an optimal surgical field, minimizing traction and allowing greater flexibility in selecting the minilaparotomy site. Conversely, ECA is technically less demanding but can be challenging in overweight patients with a short mesentery. Furthermore, unnoticed intestinal rotation during anastomosis may occur. The influence of anastomotic technique on IH rates remains poorly defined, with currently available data being limited and inconclusive [15]. While ICA seems to offer potential advantages over ECA, its overall clinical benefit is still a matter of debate. Existing meta-analyses, primarily based on retrospective studies, have produced conflicting outcomes, highlighting the need for more robust evidence to clarify this association [16–18].

This study aimed to evaluate differences in IH incidence between ICA and ECA in the setting of LRC.

## Methods

### Inclusion criteria and population under study

**Fig. 1:**
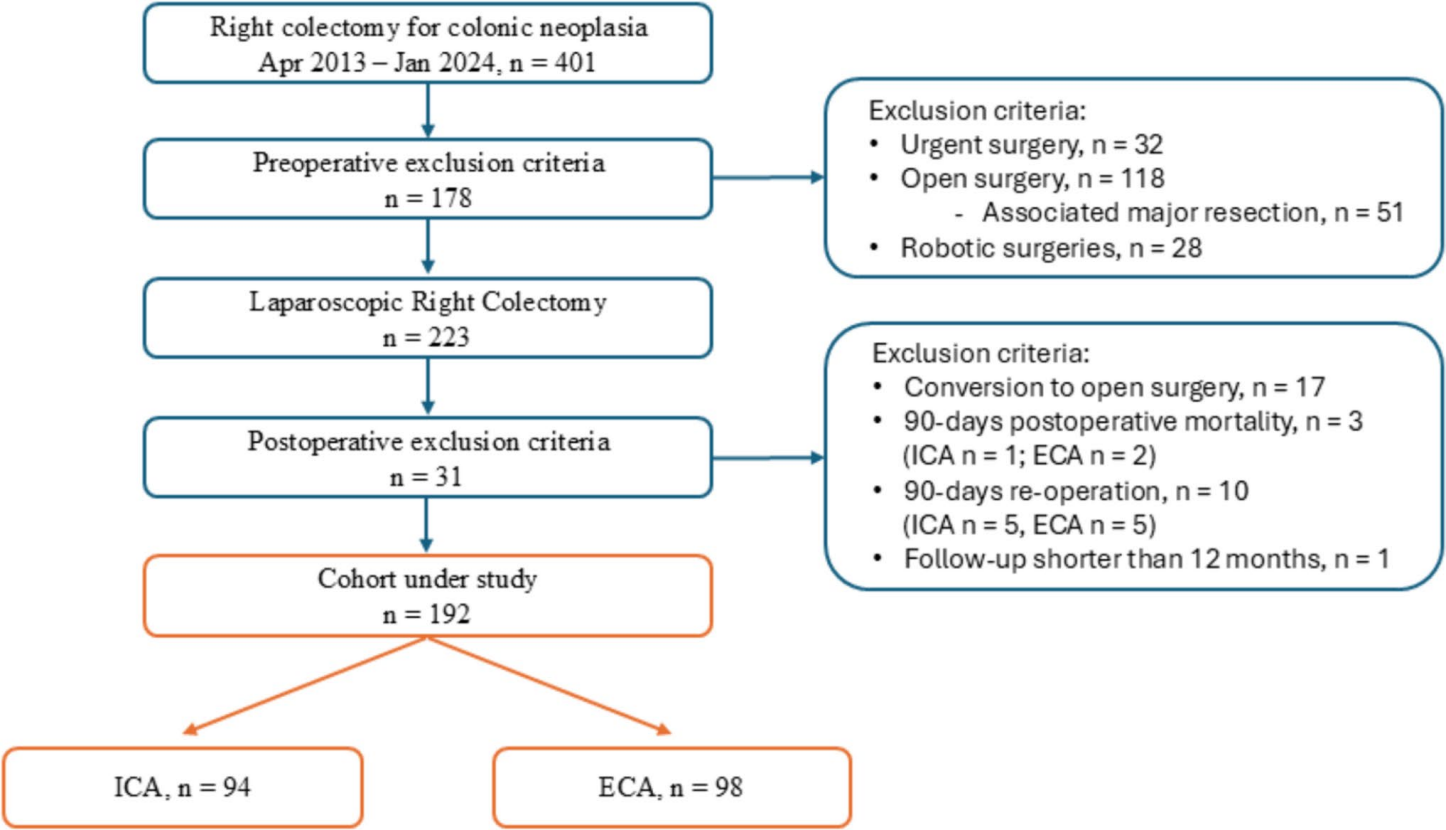
Flow diagram of patients'selection

This is a single-centre, retrospective, cohort study comparing the outcomes of ICA and ECA in patients submitted to LRC for neoplasia at the Division of General and Hepatobiliary Surgery, University of Verona Hospital Trust between April 2013 and January 2024. All consecutive patients aged 18 years or older undergoing elective LRC for colon neoplasia (i.e., adenomas not amenable of endoscopic resection, adenocarcinoma, and neuroendocrine tumor) and a minimum follow-up of 12 months were enrolled in the study. Patients who needed conversion to open surgery (n = 17) and re-operation (n = 10) were excluded from the analysis (Fig. 1).

Informed consent was obtained from all the patients, and the study was approved by the local ethics committee (No. 42763-CRINF-1034 CESC).

### Anastomosis technique

Both in ICA and ECA groups, the transection of the colon and distal ileum was performed intracorporeally after completing the dissection and vessel sectioning, and the specimen was stored in a retrieval bag. The staplers used over the years were both manual and powered (ECHELON™, ECHELON FLEX™, iDrive™ or Signia™). The transverse colon was sectioned using a 60 mm stapler with blue/purple cartridge, whilst a 60 mm stapler with white/tan cartridge was used for the terminal ileum.

In the ECA group, a transumbilical minilaparotomy was achieved enlarging the umbilical optical port. In selected cases of lean patients with long transverse mesentery, ECA was seldomly performed though a suprapubic minilaparotomy. A side-to-side isoperistaltic double-layer hand-sewn anastomosis was the preferred method, and it was fashioned after the positioning of a wound protector (Alexis® S, Applied) and specimen extraction.

In the ICA group, a side-to-side isoperistaltic stapled anastomosis was performed using an endoscopic linear stapler with white/tan cartridge and the enterotomy was closed with a double layer running suture using a barbed suture. The specimen was retrieved at the end of the procedure preferentially through a suprapubic transverse incision after the positioning of a wound protector (Alexis® S, Applied). In case of small tumors in thin patients or previous laparotomies, a transumbilical minilaparotomy was seldom used.

### Postoperative and follow-up data

Postoperative morbidity was defined as any deviation from the expected postoperative course and complications were graded according to the Clavien-Dindo classification [19]. Pathology specimens were analysed and reported according to the 8th Edition of the AJCC [20]. IHs were diagnosed during the follow-up outpatient visits through clinical examination, routine follow up CT scan, or both. Clinically, an IH was identified when a bulge or weakness in the abdominal wall at the site of a surgical incision was observed; radiologically, it was defined as a defect in the abdominal wall fascia.

### Data collection and statistical analysis

All demographic, clinical and long-term data were prospectively collected. Continuous data were analysed with the Student’s t-test or the Kruskal–Wallis test as appropriate. Categorical data were compared using the chi-square test or Fisher exact test. Continuous data were presented as means (standard deviation), or medians (IQR) depending on distribution. Categorical data were presented as frequencies. Logistic regression model was used to estimate the strength of association between IH and other factors when including into the model relevant covariates. Covariates were established following consensus among the investigators based on clinical reasoning and literature review [21–23]. Covariates were tested with univariate logistic regression and were entered in multivariable analysis if they presented a p-value < 0.2 at univariate analysis. For variables presenting collinearity, the one with the lower p-value was considered for the multivariate analysis. The analysis included the following variables: age, gender (female vs. male), body mass index (BMI), American Society of Anesthesiologists (ASA) status (≤ 2 vs. > 2), smoking habits (yes vs. no), diabetes (yes vs. no), other comorbidities potentially affecting IH (yes vs. no), preoperative anaemia (yes vs. no), previous abdominal surgery (yes vs. no), TNM tumor stage (≤ II vs. > II), postoperative chemotherapy (yes vs. no), duration of surgery, length of minilaparotomy, location of minilaparotomy (suprapubic vs. non-suprapubic), overall complications (yes vs. no), general complications (yes vs. no), pulmonary complications (yes vs. no), surgery-related complications (yes vs. no), prolonged postoperative ileus (PPOI) (yes vs. no), wound-related complications (yes vs. no), SSI (yes vs. no), type of anastomosis (ECA vs. ICA). Among other comorbidities potentially affecting the occurrence of IH, chronic pulmonary disease, chronic renal failure, malnutrition, connective tissue disorders, long-term use of steroids or immunosuppressants were considered. Preoperative anaemia (suboptimal haemoglobin level) was defined as a haemoglobin concentration of less than 130 g/L for males and less than 120 g/L for females, as defined by World Health Organization (WHO), [24]. A p value of < 0.05 was considered statistically significant. Statistical analysis was performed using SPSS software 23.0 version (IBM Corporation, Armonk, NY).

## Results

### Cohort-under study

A total of 192 patients were examined, 94 patients in the ICA group and 98 in the ECA group. Patients’ demographic and clinical data are reported in Table 1. ICA group included a greater proportion of males (60.6% vs. 44.9%; *p* = 0.031) whilst, no significant differences were demonstrated in terms of age, BMI, previous abdominal surgeries, smoking habit, diabetes, other comorbidities affecting IH rate, preoperative anaemia, and ASA status. Similarly, no difference was observed considering TNM stage.

**Table 1:** Demographic and clinical data according to study group

Data	ICA group *(n* = *94)*	ECA group *(n* = *98)*	*p* Value
Age, years (mean ± SD)	70.3 ± 11.6	68.9 ± 12.7	0.402
Male gender	57 (60.6)	44 (44.9)	**0.031**
BMI, kg/m^2^ (mean ± SD)	25.4 ± 3.9	25.7 ± 5.3	0.672
ASA status > 2	34 (36.2)	29 (29.6)	0.359
Smoking	18 (19.1)	21 (21.4)	0.723
Diabetes	20 (21.3)	18 (18.4)	0.718
Other comorbidities	10 (10.6)	19 (19.4)	0.108
Preoperative anemia	51 (54.3)	46 (46.9)	0.317
Previous abdominal surgery	48 (51.1)	45 (45.9)	0.564
TNM stage > II	33 (35.1)	33 (33.7)	0.880
Postoperative chemotherapy	23 (24.7)	22 (22.4)	0.736

*SD* standard deviation

### Surgery and postoperative data

Surgery and postoperative data are detailed in Table 2. Extent of colonic resection, lymph node count, duration of surgery, and estimated intraoperative blood loss were comparable between groups. The site of specimen extraction differed significantly, specifically transverse suprapubic incision was adopted in 80.9% of ICA cases, whilst transumbilical incision in 94.9% of ECA cases (*p* < 0.001).

**Table 2:** Surgery and postoperative data according to study group

Data	ICA group *(n* = *94)*	ECA group *(n* = *98)*	*p* Value
Extent of colonic resection			0.744
Right colectomy	89 (94.7)	94 (95.9)	
Extended right colectomy	5 (5.3)	4 (4.1)	
No. of analyzed nodes	27.8 ± 13	27.5 ± 13	0.853
Duration of surgery, mins (mean ± SD)	224 ± 69	219 ± 66	0.594
Estimated blood loss, mL (mean ± SD)	52 ± 58	56 ± 46	0.607
Length of minilaparotomy, cm (median, IQR)	5 (5–6)	6 (5–6)	0.217
Site of minilaparotomy			** < 0.001**
Transumbilical	18 (19.1)	93 (94.9)	
Suprapubic	76 (80.9)	3 (3.1)	
Other	–	2 (2)	
Postoperative complications	44 (46.8)	35 (35.7)	0.143
Severe complications	3 (3.2)	3 (3.1)	1
General complications	23 (24.5)	18 (18.4)	0.379
Pulmonary complications	3 (3.2)	5 (5.1)	0.721
Surgery-specific complications	24 (25.5)	25 (25.5)	1
Prolonged postoperative ileus	5 (5.3)	14 (14.3)	0.052
Wound hematoma or seroma	3 (3.2)	8 (8.2)	0.214
Superficial SSI	5 (5.3)	8 (8.2)	0.569
Hospital stay, d (median, IQR)	4 (4–6)	6 (4—7)	**0.003**

*SD* standard deviation, *IQR* interquartile range, *SSI*: superficial surgical site infection

Thirty-day postoperative morbidity was comparable between the two groups. Severe complications (Clavien-Dindo > 2) not requiring reoperation occurred in 3.2% of patients in the ICA group and 3.1% in the ECA group. There were no significant differences in surgery-related complications, including SSI (5.3% vs. 8.2%; *p* = 0.569) and hematoma/seroma formation (3.2% vs. 8.2%; *p* = 0.214). Similarly, the incidence of general and pulmonary complications was comparable between groups. Conversely, the incidence of PPOI was lower, approaching statistical significance, in the ECA group (14.3% vs. 5.3%; *p* = 0.052). With regards to postoperative lenght of stay (LOS), patients in the ICA group showed shorter hospital stay (Median [IQR]: 4 [4–6] days vs. 6 [4–7] days; *p* = 0.003).

### Incisional hernia

Overall, the incidence of IH was 1.1% in the ICA group and 13.3% in the ECA group (*p* = 0.001). No IH was observed at the site of minilaparotomy after ICA, whilst ECA group suffered a much higher rate of 13.3% (*p* < 0.001). The only case of IH in the ICA group occurred at the umbilical optical port site.

Logistic regression analysis is reported in Table 3. At univariate analysis, the occurrence of postoperative general complications (OR [95% CI]: 3.1 [1.0–9.4]; *p* = 0.048), the location of minilaparotomy (OR [95% CI]: 10.1 [1.3–79.2], *p* = 0.027) and the anastomosis technique (OR [95% CI]: 14.2 [1.8–111.0]; *p* = 0.011) resulted to be significant factors for IH. Given the strong association between the anastomosis technique and the location of minilaparotomy (Table 2, *p* < 0.001), only anastomosis technique was included in the multivariate model to reduce the risk of multicollinearity.

**Table 3:** Univariate and multivariate logistic regression analysis for risk factors for incisional hernia

	Univariate		Multivariate	
Covariate	OR (95% CI)	*P* Value	OR (95% CI)	*P* Value
Age	1.1 (0.9—1.1)	0.180	1.1 (0.9—1.1)	0.567
Gender (Female)	2.1 (0.7–6.5)	0.197	2.2 (0.6–8.4)	0.219
BMI	1.0 (0.9–1.1)	0.977		
ASA status (> 2)	1.6 (0.5–4.8)	0.409		
Smoking habit (yes)	0.6 (0.1–3.0)	0.563		
Diabetes (yes)	2.4 (0.8–7.8)	0.130	2.5 (0.7–9.4)	0.173
Other comorbidities (yes)	2.8 (0.8–9.8)	0.100	1.1 (0.2–4.6)	0.954
Preoperative anaemia (yes)	1.8 (0.6–5.7)	0.291		
Previous abdominal surgery (yes)	1.1 (0.4–3.2)	0.903		
TNM stage (> II)	0.5 (0.1–1.9)	0.298		
Postoperative chemotherapy (yes)	0.9 (0.2–3.3)	0.845		
Duration of surgery (yes)	1.0 (0.9–1.0)	0.962		
Length of minilaparotomy	1.0 (0.9–1.1)	0.904		
Location of minilaparotomy	10.1 (1.3–79.2)	**0.027**		
Postoperative complications (yes)	1.0 (0.3–3.1)	0.958		
General complications (yes)	3.1 (1.0–9.4)	**0.048**	4.1 (1.0–16.5)	**0.049**
Pulmonary complications (yes)	4.8 (0.9–26.3)	0.072		
Surgery-specific complications (yes)	0.5 (0.1–2.1)	0.327		
Prolonged postoperative ileus (yes)	0.6 (0.1–4.6)	0.604		
Wound-related complications (yes)	1.3 (0.2–10.9)	0.814		
Surgical site infection (yes)	1.1 (0.1–8.8)	0.954		
Type of anastomosis (ECA)	14.2 (1.8–111.0)	**0.011**	15.4 (1.9–126.9)	**0.011**

*OR* odd ratio, *CI* confidence interval.*

Multivariate analysis, adjusting for age, gender, diabetes, other chronic comorbidities, postoperative general complications, and anastomosis technique, identified postoperative general complications (OR [95% CI]: 4.1 [1.0–16.5]; *p* = 0.049) and anastomosis technique (OR [95% CI]: 15.4 [1.9–126.9]; *p* = 0.011) as significant and independent risk factors for IH development.

## Discussion

Among the advantages of MIS, the reduction in wound-related complications is one of the most well-documented [25]. The incidence of IH ranges from 3.2% to 13% following laparoscopic colorectal resections, compared to 9% to 33% after open procedures [8, 9]. The variability in the incidence is influenced by several factors, including patient characteristics, type of disease, and surgical technique.

MIS is the preferred approach for right colectomy, with laparoscopic surgery remaining the most adopted technique. However, some debate persists regarding the extent of surgical dissection as well as the optimal anastomosis technique.

The anastomotic technique used in LRC may impact IH incidence, however clear data on this correlation are lacking [26–28]. The advantages and disadvantages of ICA compared to ECA are relatively well understood, but data demonstrating overall benefit of a fully laparoscopic right colectomy remain limited [15–17]. Due to these considerations and the technical challenges associated with ICA, its adoption among surgeons remains relatively low [29].

Several studies have reported improved short-term outcomes with ICA compared to ECA. Among the most well-documented benefits there are a faster recovery of bowel function and a reduced incidence of PPOI. This is particularly relevant for overweight patients with a short mesentery, in whom ECA can be technically challenging and often requires excessive traction on the mesentery [30]. However, the increased technical complexity of ICA has been associated with longer operative times and a higher risk of anastomotic leakage [31].

In our experience, a reduction in PPOI after ICA was observed (*p* = 0.052) without a significant increase in operative time. This reduction in PPOI, though not statistically significant, contributed to a significant reduction in LOS with a median decrease of up to two days (*p* = 0.001).

In this series, no cases of anastomotic leakage were reported. This is attributable to the fact that re-operation was considered among the exclusion criteria, and in our experience, all cases of leakage underwent laparoscopic or open redo surgery. By excluding these patients, who were similarly distributed between the two groups, we believe that neither the anastomosis technique nor reoperation had a significant risk of influencing the results (Fig. 1). When analysing wound-related complications, wound hematoma and seroma and SSI were slightly in favour of ICA, though the difference was not statistically significant (Table 2).

A key advantage of ICA is the flexibility in choosing the optimal extraction site. In this series, a suprapubic incision was preferred in nearly 80% of cases, while a transumbilical minilaparotomy was reserved for low-risk patients requiring smaller incisions or those with prior midline laparotomies. Conversely, in the ECA cohort, a transumbilical minilaparotomy was used in 95% of cases (*p* < 0.001). The umbilical region is inherently vulnerable to IH due to its structural characteristics [32]. Unlike other areas of the abdominal wall, it lacks a continuous muscle layer and relies solely on the linea alba for reinforcement. This intrinsic weakness predisposes the region to IHs [33]. While the higher risk of IH in the umbilical region is well recognized, transumbilical minilaparotomy remains a quick and technically simple approach. Moreover, it represents the safest site in case of ECA fashioning [34]. In rare cases, ECA can be performed through a suprapubic minilaparotomy, typically in lean patients with a long and well-mobilized transverse colon, as it was in our experience.

In our series, no IH was observed in the ICA group, whereas the incidence in the ECA group was higher than 13% (*p* < 0.001). This difference is in line with previous anatomical and pathophysiological considerations.

Among the factors that may contribute to the development of IH, some are related to patient characteristics and the underlying disease, such as age, BMI, smoking habits, diabetes, other chronic diseases, long-term use of steroids, and tumor stage. Additional factors related to the operative technique and postoperative course are supposed to play a role, as they directly affect tissue integrity and healing. These include the type of surgery, incision length and location, wound complications, and both general and surgery-specific complications.

Univariate analysis identified the occurrence of postoperative general complications (OR [95% CI]: 3.1 [1.0–9.4]; *p* = 0.048), minilaparotomy site other than suprapubic (OR [95% CI]: 10.1 [1.3–79.2]; *p* = 0.027), and ECA (OR [95% CI]: 14.2 [1.8–111.0]; *p* = 0.011) as significant risk factors for the development of IHs.

Due to collinearity between the location of minilaparotomy and the anastomosis technique, only the anastomosis technique was included in the multivariable analysis, as it represented the primary focus of our study and was further justified by its higher odds ratio. Logistic regression analysis confirmed the occurrence of general complications (OR [95% CI]: 4.1 [1.0–16.5]; *p* = 0.049) and ECA (OR [95% CI]: 15.4 [1.9–126.9]; *p* = 0.011) as the sole independent risk factors for IH.

These findings emphasize the importance of selecting the most appropriate surgical technique to optimize patient outcomes and minimize both short- and long-term complications. IH, as a late postoperative complication, not only significantly impairs patients’ quality of life and physical function but also imposes a substantial financial burden on both patients and the healthcare system [7]. For these reasons, its prevention should be regarded as a key objective of MIS.

In an era where MIS has long been transforming surgical paradigms by reducing postoperative complications and physiological stress, the occurrence of IH should be regarded as a real failure of treatment. Various strategies have been proposed to increase the adoption of ICA, including the use of robotic surgery [35]. In our opinion, the significant reduction in IH risk associated with ICA compared to ECA should be considered as a primary factor driving the adoption of this technique, provided that a comparable anastomotic complication rate can be achieved.

This study has some limitations. First, its retrospective design may have led to an underestimation of IH occurrence or the presence of risk factors, potentially affecting the accuracy of our analysis. However, all patients were personally followed up by the surgical team through regular outpatient clinical visits and radiological examinations as part of their oncological follow-up. This ensured a precise assessment of IH occurrence, significantly mitigating the risk of underestimation. Second, the limited sample size may have reduced the ability to detect the role and significance of certain risk factors, particularly those that were less frequent in the cohort. Nevertheless, the substantial difference in IH occurrence between the ECA and ICA groups and the ORs shown at univariate and multivariable analysis for ECA group are sufficient to support the study’s primary objective.

Despite these limitations, this study has several strengths. The surgical technique was highly standardized over the time of study, with no differences between the ICA and ECA groups except for anastomosis fashioning. All surgical steps before anastomosis, including mesentery and bowel sectioning, were performed laparoscopically, ensuring comparable results in terms of dissection, as demonstrated by the similar number of analysed lymph nodes, and wound characteristics, as indicated by the length of the minilaparotomy. Additionally, the ICA and ECA groups did not exhibit significant differences in clinico-pathological characteristics or surgical data. Therefore, no major confounding factors are likely to have influenced the results of our analysis. Also, the length of follow-up ensures the detection of IH potentially occurring long after surgery, strengthening the reliability of our findings. Finally, to the best of our knowledge, our experience is one of the few studies that specifically examines IH occurrence after LRC, and the sole that analyses the anastomosis technique while considering potential confounding factors.

## Conclusions

The incidence of IH at the minilaparotomy site is significantly reduced after ICA compared to ECA, due to the increased flexibility in selecting the optimal site for specimen extraction. This finding was further confirmed by logistic regression analysis, which identified ICA as a significant and independent protective factor against IH. However, larger cohorts are needed to validate these results and overcome the study's limitations, particularly its retrospective design and limited sample size.

## Data Availability

The data are available in a personal dataset (SPSS file) and can be provided upon request to the corresponding author.
